# Comparative evaluation of bracket bond failure rates of a novel non-primer adhesive with a conventional primer-based orthodontic adhesive – a pilot study

**DOI:** 10.34172/joddd.2023.36953

**Published:** 2023-04-03

**Authors:** Kavitha Ramsundar, Ravindra Kumar Jain, Nivethigaa Balakrishnan, Bommireddy Vikramsimha

**Affiliations:** ^1^Department of Orthodontics, Saveetha Dental College, Saveetha Institute of Medical and Technical Sciences, Saveetha University, Chennai, India; ^2^Department of Public Health Dentistry, Sibar Institute of Dental Sciences, Guntur, India

**Keywords:** Adhesives, Composite resins, Dental bonding, Orthodontic brackets

## Abstract

**Background.:**

Bonding is an important step in fixed orthodontic therapy and evaluation of bracket bond failures while using different bonding systems is required. The aim of the present study was to evaluate and compare bracket failure rates of a novel no primer adhesive with conventional primer-based orthodontic adhesives.

**Methods.:**

This split mouth study was conducted among fifteen patients who underwent therapy with fixed orthodontic appliances using metal brackets. Total of 300 brackets were bonded and the bracket bond failure rates were assessed at the end of 3 months. The difference in bond failure rates between the two groups were assessed in different teeth. Descriptive statistics and chi-square test was performed.

**Results.:**

Evaluation of bracket bond failure rates showed a higher incidence of bond failures in the group bonded with the primerless adhesive (6.3%) compared to conventional adhesive (2.3%) but there was no statistically significant difference (*P*>0.05). No intergroup difference was found in the bracket failure rates of individual teeth (*P*>0.05).

**Conclusion.:**

Higher incidence of bond failures were noted with brackets bonded with primerless adhesive when compared to primer-based adhesive but no significant difference was noted over a period of 3 months. Mandibular canine and premolars had a higher bracket failure rate with no significant difference between the adhesives.

## Introduction

Bonding is a crucial step in fixed orthodontic therapy. Enamel etching followed by priming and use of resin adhesives is considered as the most widely accepted method for facilitating adhesion of the orthodontic bracket to the tooth.^1^ Primers moisten and penetrate the enamel, protecting the etched tooth surface from plaque and food residues that induce decalcification.^2^ Despite its benefits, skipping the priming process would save chairside time, enable the clinician to maintain a dry working field, and prevent bond failure because of the elimination of moisture contamination.^3^ Clinical bonding has been found to be successful with shear bond strengths even in low ranges of 6-8 Mpa.^4,5^ Some studies have reported no change in the adhesion of brackets to enamel regardless of whether the surface was primed beforehand or not, which has sparked debate among orthodontists.^2,6^

Debonding can occur immediately after the orthodontic bracket is placed in a clinical setting due to stresses induced by the orthodontic technique, adhesive cement contraction, or regular oral processes like mastication.^7,8^ Many a researchers have published papers on the bond failure rates of various types of adhesive systems.^9-11^ Two-stage conventional systems such as Transbond XT and a single stage self-etching primer such as Transbond Plus were the most widely employed materials in prior investigations.^12-18^

Tang et al reported successful bonding of metal brackets without the use of a liquid resin on the enamel surface before using a self-cure adhesive(two-paste systems) under ideal circumstances.^19^ In-vitro studies have reported sufficient shear bond strength of brackets bonded with primerless adhesives to enamel when compared to brackets bonded with primer based adhesives.^20,21^ But there is a paucity of literature on clinical trials evaluating the bracket failure rates with primerless adhesives. Hence, the present study was undertaken to evaluate and compare bracket failure rates of a novel primerless adhesive with a conventional primer based orthodontic adhesive.

## Material and Methods

 This pilot split mouth randomized study was conducted in a university hospital setting. The present study was performed at the Department of Orthodontics, Saveetha Dental college and hospital, Chennai.

 Fifteen patients who required corrective orthodontic treatment but had never undergone any form of orthodontic treatment before were selected and included in the study. Subjects in the age range of 18-35 years with either Angle’s class I or class II malocclusion with full complement of erupted permanent teeth and no occlusal interferences were included. Patients who required orthognathic surgeries or tooth extractions for malocclusion correction, those who had prosthetic dental crowns, dental restorations, hypoplastic enamel surfaces, missing teeth, and craniofacial anomalies were excluded from the study.

 The subjects were explained about the study protocol and written informed consent was obtained before starting orthodontic treatment. Appropriate instructions regarding oral hygiene and self-care of orthodontic appliances were issued to all the patients prior to the start of the study. The selected patients underwent therapy with fixed orthodontic appliances using 0.022*0.028 slot 3M Unitek Gemini brackets. Bonding of the brackets were carried out from the premolar to premolar in the maxillary and mandibular teeth with standard protocols by an experienced operator (final year postgraduate student with an experience of more than 50 full arch bonding procedures) not involved in the study. Bonding with the different adhesive systems was done in quadrants randomly by ensuring that enamel treatments were evenly distributed across the right and left sides. Group A - teeth bonded with Orthofix SPA (152) and group B-teeth bonded with Transbond XT (148). Oral prophylaxis was performed prior to bonding procedure with a low-speed handpiece using rubber cup and pumice, followed by rinsing with water and enamel etching was done with 37% orthophosphoric acid (AXOTECH) for 15-20 seconds. Isolation was achieved using cheek retractors, suction and cotton roll. For bonding, different steps were followed for both adhesives, application of bonding agent for Orthofix SPA adhesive was not required because of their self-priming nature. The adhesives were cured with a curing light unit (Ortholux Luminous, 3M) for 6 secs on both sides as recommended by the manufacturer. Bonding was completed in both arches on the same visit and then the initial archwire (0.014 inch NiTi) for leveling was inserted in both the upper and lower arches on the same day. Patients were given instructions on proper maintenance of the orthodontic appliances and educated regarding the avoidance of foods and habits that could exert damage to the bonded brackets. Patients were instructed to check for debonded brackets on a daily basis. In the event of debonding of a bracket, patients were instructed to record the date of debonding and bring the debonded bracket to the hospital as soon as possible. Re-evaluation of all the patients included in the study was carried out once every four weeks and the bond failure rates for the first 3 months were calculated.

###  Statistical analysis

Based on the bracket failures reported a chart was made and statistical analysis was performed using version 26.0 SPSS Inc., Chicago, IL, USA. Categorical data was expressed using percentages. Chi square test was used to compare the debonding incidents among various teeth. *P* value of less than 0.05 was considered to be significant.

## Results

This study included a total of 15 patients (12 males and 3 females) with mean age of 22 ± 2 years and a total of 300 teeth were bonded (Table 1).

**Table 1:** Frequency distribution data of included teeth

**Groups**	**No. teeth bonded (T0)**	**No. of teeth ** **Debonded (T1)**	**No. of teeth debonded in maxillary arch (T1)**	**No. of teeth debonded in mandibular arch (T1)**
Group A	152 (50%)	19 (6.3%)	10 (3.33%)	9 (3%)
Group B	148 (49.3%)	7 (2.3%)	2 (0.6%)	5 (1.6%)

The overall bracket failures observed in this study was 8.6%. In the maxillary arch for group A, the highest incidence of debonding was noted in 24 (25%) followed by 12 (16.7%). In group B debonding was reported in 24 (14.3%) and 25 (14.3%). However, there was no significant inter-group difference noted (*P* > 0.05) (Table 2).

**Table 2:** Comparison of bracket failures in the Maxillary arch

**Tooth number**	**Groups**	**Debonded** **No. (%)**	**Un-debonded** **No. (%)**	***P*** ** value**
11	Group A	0	12 (100)	-
	Group B	0	3 (100)	
12	Group A	2 (16.7)	10 (83.3)	0.44
	Group B	0 (0)	3 (100)	
13	Group A	1 (8.3)	11 (91.7)	0.60
	Group B	0 (0)	3 (100)	
14	Group A	1 (8.3)	11 (91.7)	0.60
	Group B	0 (0)	3 (100)	
15	Group A	0	12 (100)	--
	Group B	0	3 (100)	
21	Group A	1 (12.7)	7 (87.5)	0.33
	Group B	0	7 (100)	
22	Group A	1 (12.7)	7 (87.5)	0.33
	Group B	0	7 (100)	
**23**	Group A	1 (12.7)	7 (87.5)	0.33
	Group B	0	7 (100)	
**24**	Group A	2 (25)	6 (75)	0.60
	Group B	1 (14.3)	6 (85.7)	
**25**	Group A	1 (12.5)	7 (87.5)	0.91
	Group B	1 (14.3)	6 (85.7)	

In the mandibular arch for both groups the highest incidence of debonding was noted in 45 (20%). However, there was no significant difference noted on statistical analysis (*P* > 0.05) (Table 3).

**Table 3:** Comparison of bracket failures in the mandibular arch

**Tooth number**	**Groups**	**Debonded** **No. (%)**	**Un-debonded** **No. (%)**	***P*** ** value**
31	Group A	0	5 (100)	--
	Group B	0	10 (100)	
32	Group A	0	5 (100)	--
	Group B	0	10 (100)	
33	Group A	1 (20)	4 (80)	0.14
	Group B	0 (0)	10 (100)	
34	Group A	1 (20)	4 (80)	0.14
	Group B	0 (0)	10 (100)	
35	Group A	1 (20)	4 (80)	0.59
	Group B	1 (10)	9 (90)	
41	Group A	1 (20)	4 (80)	0.59
	Group B	1 (10)	9 (90)	
42	Group A	1 (20)	4 (80)	0.14
	Group B	0 (0)	10 (100)	
43	Group A	1 (20)	4 (80)	0.14
	Group B	0 (0)	10 (100)	
44	Group A	1 (20)	4 (80)	0.14
	Group B	0 (0)	10 (100)	
45	Group A	2 (20)	5 (80)	1.00
	Group B	3 (20)	5 (80)	

## Discussion

Orthodontic bracket failure is a major concern during fixed orthodontic treatment and can affect the overall success of the treatment. The detachment of the brackets during orthodontic corrective procedures can lead to an increase in the duration of the treatment, and can increase the cost of overall treatment.^22^ The ideal bonding of the bracket to the enamel should be adequate to ensure a stable bracket position throughout treatment and bracket failure.^23^ In the present study evaluation of the bracket failure rate of a primerless adhesive (Orthofix SPA) was compared with a conventional primer-based adhesive (Transbond XT). An overall higher bracket failure rate (6.3%) was observed in teeth bonded with primerless adhesive than conventional adhesive. No significant individual teeth differences in the bracket failure rate between the two adhesives were noted.

Previous in vivo studies have reported on bracket failure rate of conventional primer based composites. Orthofix is a conventional primer based adhesive and Ortho fix SPA is a novel primerless adhesive system by the same company. A study by Samantha et al. reported on bracket failure rate comparison between two primer based adhesives (Orthofix and Transbond XT) and found a higher bracket bond failure rate with Orthofix, but there was no statistical significance.^24^ Rai et al in 2015 reported a study where bond failure rates were assessed between Transbond XT with and without primer. In this study the same adhesive was used with and without primer in alternate subjects. Teeth bonded with non-priming adhesive debonded more often than the ones bonded with primers and the difference was statistically significant.^25^ Likewise in the present study a higher failure rate was noted with primerless adhesive but the difference was not statistically significant. Only difference between the above mentioned study and the present study was that the same adhesive system with and without primer was used in their study. Though bonding adhesive play a major role in preventing the failure of bonded brackets, various other factors such as diet, patient compliance, tooth morphology, technique of bonding etc. are also influential in determining the overall survival time of the bonded brackets.^22^ Transbond XT is a light cured Bis-GMA based composite adhesive that is widely used in orthodontics for bonding.

Time duration considered for the evaluation of the bracket failure is a major factor determining the prevalence of bracket failure rates. Differences in duration of evaluation were noted among various studies in reporting the prevalence of bracket failures.^16,26,27^ In the present study a follow up period of 3 months was considered to evaluate the incidents of bracket failures. Various other studies by Ireland JA et al. and Elekdag-Turk et al considered evaluation for bracket failures by a follow up of 6 months.^26,27^ While Krishnaswamy et al considered a follow up of 15 months.^28^ In this present study higher bracket failures were noted in mandibular Canines and premolars. Most studies have reported premolars as the high-risk site for debonding of brackets.^29,30^ In this present study there was no difference in the bracket failure rate of premolars between the two groups. This study is bound to limitations such as sample size calculation was not done, other patient related factors such as the type of malocclusion, growth patterns and muscle function were not taken into account. Further studies with a long term follow up evaluating the bracket failure rate of various adhesive systems considering the various factors are needed

## Conclusion

 Bracket debonding incidents were reported with both the adhesive systems used in this study. Even though more bracket failures were evident with primerless adhesive when compared to primer-based adhesive no statistically significant difference was noted. Mandibular canine and premolars had a higher bracket failure rate but there was no significant difference.

## Acknowledgments

 We would like to express our sincere gratitude to our research supervisor Dr. Ravindra Kumar Jain, professor, Department of Orthodontics, Saveetha Dental College and Hospital, Chennai, for his guidance and encouragement with our work in all stages. We would also like to acknowledge Dr. Nivethigaa B and Dr. Bommireddy Vikramsimha for their contribution to this study.

## Competing Interests

 No conflicts of interest.

## Ethical Approval

 The study protocol was approved by the Ethical Committee of SIMATS, Saveetha Dental College and Hospital, Chennai, India (IRB number is IHEC/SDC/ORTHO-2007/21/652).

## Funding

 No funding.
